# Redox Regulation of the Quorum-sensing Transcription Factor AgrA by Coenzyme A

**DOI:** 10.3390/antiox10060841

**Published:** 2021-05-25

**Authors:** Jovana Baković, Bess Yi Kun Yu, Daniel Silva, Maria Baczynska, Sew Yeu Peak-Chew, Amy Switzer, Lynn Burchell, Sivaramesh Wigneshweraraj, Muralidharan Vandanashree, Balasubramanian Gopal, Valeriy Filonenko, Mark Skehel, Ivan Gout

**Affiliations:** 1 Department of Structural and Molecular Biology, University College London, London WC1E 6BT, UK; jovana.bakovic.16@ucl.ac.uk (J.B.); bess.yu.15@ucl.ac.uk (B.Y.K.Y.); danielsilvaslb01@gmail.com (D.S.); mb01554@surrey.ac.uk (M.B.); 2 MRC Laboratory of Molecular Biology, Cambridge Biomedical Campus, Cambridge CB2 0QH, UK; spc@mrc-lmb.cam.ac.uk (S.Y.P.-C.); mskehel@mrc-lmb.cam.ac.uk (M.S.); 3 Section of Microbiology, Faculty of Medicine and MRC Centre for Molecular Bacteriology and Infection, Imperial College London, London SW7 2AZ, UK; amy.switzer09@imperial.ac.uk (A.S.); l.burchell@imperial.ac.uk (L.B.); s.r.wig@imperial.ac.uk (S.W.); 4 Molecular Biophysics Unit, Indian Institute of Science, Bangalore 560012, India; vandanashree@iisc.ac.in (M.V.); bgopal@iisc.ac.in (B.G.); 5 Department of Cell Signaling, Institute of Molecular Biology and Genetics, 143 Kyiv, Ukraine; filonenko@imbg.org.ua

**Keywords:** *Staphylococcus aureus*, quorum-sensing, AgrA, oxidative stress, protein CoAlation, transcriptional regulation

## Abstract

*Staphylococcus aureus* (*S. aureus*) is an aggressive opportunistic pathogen of prominent virulence and antibiotic resistance. These characteristics are due in part to the accessory gene regulator (*agr*) quorum-sensing system, which allows for the rapid adaptation of *S. aureus* to environmental changes and thus promotes virulence and the development of pathogenesis. AgrA is the *agr* system response regulator that binds to the P2 and P3 promoters and upregulates *agr* expression. In this study, we reveal that *S. aureus* AgrA is modified by covalent binding of CoA (CoAlation) in response to oxidative or metabolic stress. The sites of CoAlation were mapped by liquid chromatography tandem mass spectrometry (LC–MS/MS) and revealed that oxidation-sensing Cys199 is modified by CoA. Surface plasmon resonance (SPR) analysis showed an inhibitory effect of CoAlation on the DNA-binding activity, as CoAlated AgrA had significantly lower affinity towards the P2 and P3 promoters than non-CoAlated AgrA. Overall, this study provides novel insights into the mode of transcriptional regulation in *S. aureus* and further elucidates the link between the quorum-sensing and oxidation-sensing roles of the *agr* system.

## 1. Introduction

Pathogenic bacteria employ a variety of mechanisms to overcome the host’s immune response and to spread infections. The most-studied and relevant mechanism is arguably the quorum-sensing system, which detects cell density through cell-to-cell communication mechanisms and consequently regulates the expression of specific genes responsible for immune evasion and virulence. Numerous processes are controlled by quorum-sensing in bacteria, such as sporulation, gene transfer, biofilm formation, and virulence factor secretion [1]. 

*Staphylococcus aureus* (*S. aureus*) is a Gram-positive bacterium normally found on human skin and mucous membranes. It is an opportunistic human pathogen that causes serious infections that can lead to acute and chronic illnesses and have life-threatening consequences [2]. The accessory gene regulator (*agr*) quorum-sensing system allows for the rapid adaptation of *S. aureus* to environmental changes and thus promotes virulence and the development of pathogenesis [3]. This two-component system induces the transcription of *rnaII* and *rnaIII* via the P2 and P3 promoters, respectively. The P2 promoter governs the expression of the *agr* operon, which contains four genes: *agrA, agrB*, *agrC*, and *agrD*. AgrA is the response regulator that binds to both P2 and P3 promoters and upregulates the transcription of *agr* and *rnaIII*, respectively. It is activated by AgrC, a transmembrane histidine kinase that is activated by autophosphorylation upon binding to the autoinducing peptide (AIP). AgrB is a transmembrane endopeptidase that cleaves the C-terminus of the AIP precursor (encoded by *agrD*), introduces a thiolactone bond between the C-terminus and an internal cysteine of AIP, and exports active AIP into the extracellular space. Indeed, AgrC and AgrA are two components of a signal transduction pathway that is activated in the late log phase of bacterial growth when the concentration of extracellular AIP is high. Increased transcription from the P3 promoter by AgrA activates RNAIII expression (the *agr* effector molecule), which itself regulates the expression of several genes responsible for virulence and immune evasion. As a result, the expression of cell surface components decreases and the transcription of virulence factors (such as hemolysins and TSS toxin-1) increases, which allows bacteria that have reached high densities to acquire more nutrients from the host and spread the infection further [3]. In addition to AIP, the *agr* system can also be activated in response to extracellular stimuli such as glucose concentration and pH, as well as transcriptional regulators SarA and SrrAB [4].

The *agr* quorum response was shown to be sensitive to oxidizing conditions, mediated by an intramolecular disulfide switch in the DNA-binding domain of AgrA [5]. AgrA contains two structural domains that mediate its transcriptional activity: the response regulator and a DNA-binding (LytTR) domain [6]. Cysteine (Cys) 199 was identified as crucial for oxidation sensing and the formation of a disulfide bond with Cys228 upon oxidative stress, which inhibits AgrA binding to DNA [5]. It has been proposed that the formation of an intramolecular disulfide in AgrA during oxidizing conditions induces a conformational change in the DNA-binding domain, leading to a steric interference and the dissociation of AgrA from DNA.

Cysteine residues on proteins are well-known targets of various oxidative post-translational modifications (oxPTMs), including S-thiolation, nitrosation, acetylation, and persulfhydration, among others. A range of enzymes, receptors, and transcription factors are regulated by oxPTMs [7]. Protein glutathionylation (the thiolation of cysteine residues by glutathione (GSH)) is the most studied form of S-thiolation and occurs in response to oxidative, nitrosative, or metabolic stress. It has a variety of functions, including protecting protein cysteines from irreversible overoxidation to the sulfonic forms [8]. Protein glutathionylation was shown to modulate regulatory interactions, DNA binding, and transcriptional activities of many prokaryotic transcriptional regulators that are involved in bacterial redox regulation and the adaptation to stresses [9,10]. GSH is produced in all Gram-negative bacteria, but not in Gram-positive *Firmicutes* (including *Bacillus* and *Staphylococcus*) and *Actinomycetes* species that synthesize bacillithiol (BSH) and mycothiol, respectively [8]. In *Bacillus subtilis*, bacillithiolation was found to regulate the activity of the redox-sensing OhrR repressor [11,12], whereas mycothiolation of several key enzymes was also described in *Actinomycetes* [13].

Coenzyme A (CoA) is another major thiol produced in all living cells by enzymatic conjugation of ATP, pantothenate (vitamin B5), and cysteine [14,15]. CoA and CoA thioesters participate in diverse anabolic and catabolic pathways, including the citric acid cycle, fatty acid biosynthesis and oxidation, amino acid metabolism, and isoprenoid and peptidoglycan biosynthesis. The antioxidant function of CoA has been recently reported in both eukaryotic and prokaryotic cells [16,17]. The development of novel research tools and methodologies has allowed the identification and characterization of CoA modified proteins (CoAlation) in vitro and in cell-based and animal models [14,18,19]. Protein CoAlation was found to be a widespread and reversible oxPTM [20,21]. To date, over 2100 CoAlated proteins have been identified by the developed mass spectrometry-based methodology in mammalian cells and bacteria exposed to oxidative or metabolic stress. CoAlation was found to modulate the activity and conformation of modified proteins, and protect key cysteine residues from overoxidation [18,19]. Bioinformatics analysis of CoAlated proteins in mammalian cells revealed that the majority (over 65%) are involved in metabolic processes. In contrast to mammalian cells, numerous transcription factors and regulators were found to be CoAlated in *S. aureus* and *Bacillus megaterium* (*B. megaterium*) exposed to oxidative stress [17]. The *agr* quorum-sensing system response regulator, AgrA, was one of them. 

In this study, we report covalent modification of AgrA by CoA in vitro and in vivo, as well as the consequent regulation of its DNA-binding activity. External challenges in the form of oxidative stress and nutrient deprivation were found to induce AgrA CoAlation. The modified cysteines residues were mapped by liquid chromatography tandem mass spectrometry (LC–MS/MS) to Cys6 and Cys199, the latter of which is located in the LytTR DNA-binding domain. Furthermore, surface plasmon resonance (SPR) showed that in vitro CoAlation of recombinant AgrA inhibits its binding to both the P2 and P3 promoters. On the basis of these findings, we propose that CoAlation of AgrA at Cys199 under oxidative or metabolic stress modulates its DNA-binding activity and may create a binding motif for the formation of novel regulatory complexes implicated in the oxidative stress response. CoAlation of AgrA thus suggests an elaborate mechanism to regulate AgrA activity that synchronizes inputs from diverse stress response pathways to calibrate quorum sensing with other environmental or intracellular stimuli. 

## 2. Materials and Methods

### 2.1. Reagents and Chemicals

All reagents and chemicals were obtained from Sigma-Aldrich unless stated otherwise.

### 2.2. Bacterial Growth Conditions, SaAgrA Overexpression and Treatmeants

*S. aureus (MRSA: DSM11729)* and *Escherichia coli (E. coli*) BL21 (DE3) cells transformed with pET28b(+)/ His-*Sa*AgrA were cultured overnight in Luria–Bertani (LB) medium. The overnight cultures were diluted 1:100 in the same medium and incubated until the optical density at 600 nm reached 0.7 (OD_600_ = 0.7). For 6xHis-*Sa*AgrA overexpression, pET28b(+)/His-*Sa*AgrA transformed *E. coli* cells were induced with 0.1 mM isopropyl-β-D-1-thiogalactopyranoside (IPTG) for 20 min at 37 °C. To induce oxidative stress, cells were treated with or without oxidizing agents for 30 min at 37 °C.

For glucose-deprivation induced stress, *E. coli* BL21 (DE3) cells transformed with pET28b(+)/His-*Sa*AgrA were induced with IPTG, harvested by centrifugation, resuspended in M9 minimal medium without glucose to remove the source of carbohydrate, and cultured at 37 °C for 30 min. For recovery experiments, starved cells were harvested and resuspended in M9 media supplemented with 20 mM glucose and incubated at 37 °C for 30 min or 60 min.

For nitrogen starvation experiments, *E. coli* BL21 (DE3) cells transformed with pET28b(+)/His-*Sa*AgrA were grown in 10 mM NH_4_Cl (for overnight cultures) or 3 mM NH_4_Cl Gutnick minimal medium (for nitrogen starvation experiments), consisting of 33.8 mM KH_2_PO_4_, 77.5 mM K_2_HPO_4_, 5.74 mM K_2_SO_4_, and 0.41 mM MgSO_4_, supplemented with Ho-LE trace elements and 0.4% (wt/vol) glucose, using NH_4_Cl as the sole nitrogen source. NH_4_Cl concentration in the media was determined using the Aquaquant ammonium quantification kit (Merck Millipore), according to the manufacturer’s instructions. The time when the ammonium ran out ([ammonium] < 0.000625 mM) in the growth medium was used as a starting point for 30 min and 60 min incubation at 37 °C for the induction of the nitrogen starvation stress. For recovery experiments, starved cells were harvested by centrifugation and resuspended in 10 mM NH_4_Cl supplemented-Gutnick minimal medium and incubated for 30 min or 60 min at 37 °C.

### 2.3. Lysis of Cells, Protein Extraction and Affinity Purification 

To extract proteins, harvested *E. coli* and *S. aureus* cells were resuspended in cell lysis buffer containing 20 mM Tris–HCl (pH 7.5), 50 mM NaCl, 50 mM NaF, 5 mM Na_4_P_2_O_7_, 0.5 mg/mL lysozyme, 100 mM *N*-Ethylmaleimide (NEM), and a cocktail of protease inhibitors (Roche). Sodium dodecyl sulfate (SDS) was added (1% final), and the homogenate was sonicated (30s on, 20 s off, 5 cycles) at 4 °C to reduce viscosity before centrifuging at 21,000× *g* for 10 min at 4 °C. The supernatant was collected and samples were boiled in 1X non-reducing SDS loading buffer (63 mM Tris HCl pH 8.0, 10% glycerol, 2% SDS, 0.0025% bromophenol blue) for 5 min before SDS-PAGE analysis and Western blotting (WB). 

The affinity purification of 6xHis-*Sa*AgrA from *E. coli* cells in oxidative stress and carbon or nitrogen deprivation experiments was carried out by incubating each sample with 10 µL bead volume of Ni^2+^ nitrilotriacetic acid (Ni-NTA) Sepharose beads for 30 min at 4 °C. Beads were washed three times with wash buffer (20 mM Tris HCl (pH 7.5), 50 mM NaCl, 50 mM NaF, 5 mM Na_4_P_2_O_7_) by centrifugation at 1000× *g* (4 °C, 2 min each). Beads were boiled in 1X non-reducing SDS loading dye for 5 min before analysis via WB. 

### 2.4. Western Blot (WB) Analysis

Samples of bacterial extracts containing 30–40 µg of proteins, or total protein samples eluted from Ni-NTA beads were separated by SDS–polyacrylamide gel electrophoresis (PAGE) on 4–20% Mini-PROTEAN TGX Precast Gels (Bio-Rad Laboratories, Hercules, CA, USA). Separated proteins were transferred from the gel to a low-fluorescence polyvinylidene fluoride membrane (Bio-Rad Laboratories), which was then blocked with Odyssey blocking buffer (LI-COR Biosciences, Lincoln, NE, USA). Mouse monoclonal anti-CoA antibody (0.17 µg/mL, generated as described previously [20]) and rabbit polyclonal anti-AgrA (WB dilution 1:250, Eurogentech, Liège, Belgium) were diluted in Odyssey blocking buffer and incubated with the membrane for 2h at RT or overnight at 4 °C. Immunoreactive protein bands were visualized using Alexa Fluor 680 goat anti-mouse IgG H&L (WB dilution 1:10,000, Life Technologies, Carlsbad, CA, USA) and IRdye 800 CW goat anti-rabbit IgG H&L (WB dilution 1:10,000, LI-COR Biosciences) on the Odyssey infrared imaging system (Odyssey Scanner CLx and Image Studio Lite software, LI-COR Biosciences). For quantitative analysis, the band intensity values for CoAlated *Sa*AgrA were obtained from anti-CoA or anti-AgrA WB through Image Studio Lite (Ver 5.2). The anti-CoA band intensities were normalized against the corresponding anti-AgrA band intensities for each WB. The mean fold increase in CoAlation signal was calculated by comparison to respective controls. For statistical analysis, a ratio paired, one-tailed Student’s *t*-test was used with GraphPad Prism (Version 9.1.1). Statistical significance was established for *p* < 0.05, and the statistical variability was estimated with the standard error of the mean (SEM).

### 2.5. Sample Preparation for Liquid Chromatography Tandem Mass Spectrometry (LC-MS/MS)

Proteins from prepared cell lysates of diamide-treated *S. aureus* cells were precipitated with 90% methanol. The protein pellet was resuspended in 50 mM ammonium bicarbonate (Ambic, pH 7.8) supplemented with 6.4 mM iodoacetamide (IAM), digested with endoproteinases Lys C and trypsin (sequencing grade, Promega, Madison, WI, USA) and heat inactivated at 99 °C, 10 min. CoAlated peptides were then immunoprecipitated with anti-CoA antibody cross-linked to Protein G Sepharose. Immunoprecipitated peptide mixtures were eluted with 0.1% trifluoroacetic acid (TFA), dried down completely in a SpeedVac and resolubilized in 20 µL of 50 mM Ambic and treated with 1.7 µg Nudix 7 phosphatase in the presence of 5 mM MgCl_2_ at 37 °C for 20 min. Then the samples were acidified, desalted with a C18 Stage tip that contained 1.5 µL of Poros R3 resin and partially dried in a SpeedVac. Desalted peptides were further incubated for 45 min with 30 µL of Phos-Select IMAC resin (Sigma) in 100 µL of 30% MeCN, 0.25 M acetic acid (loading solution) for enrichment. Beads were washed four times with loading solution and CoAlated peptides were eluted twice with 500 mM imidazole (pH 7.6) and once with 30% MeCN/500 mM imidazole (pH 7.6). Before mass spectrometry analysis, CoAlated peptides were acidified, dried, desalted and partially dried using a SpeedVac. LC-MS/MS analysis and identification of CoAlated peptides from diamide-treated *S. aureus* cells were carried out as previously described in [17].

### 2.6. Expression and Purification of Recombinant 6xHis-SaAgrA Protein

The *S. aureus agrA* coding sequence was cloned into the pET28b expression vector, and the recombinant protein was overexpressed in *E. coli* Rosetta (DE3) pLysS cells [21]. Briefly, cells were grown in LB at 37 °C to an optical density of 0.5 at 600 nm (OD_600_). Subsequently, the expression of 6xHis-*Sa*AgrA was induced with 0.5 mM IPTG. The induced cells were grown for 16 h at 18 °C and harvested by centrifugation at 1000× *g*. The harvested cells were suspended in a buffer containing 20 mM HEPES (pH 7.6), 300 mM KCl, 10% glycerol, and 2 mM phenylmethylsulfonyl fluoride (PMSF). After sonication (3 s on, 5 s off, 3 min, 2 cycles), the cell debris was separated by centrifugation at 8000× *g* for 45 min at 4 °C. The cell-free lysate was then incubated with Ni-NTA Sepharose beads (Sigma-Aldrich, Inc., St. Louis, MO, USA) for 45 min at 4 °C. The bound protein was eluted in a buffer containing 20 mM HEPES (pH 7.6), 300 mM KCl, 10% glycerol, and 250 mM imidazole. The partially purified protein fractions were concentrated and loaded onto a Sephacryl S-200 (HiPrep 16/60) column (GE Healthcare, Chicago, IL, USA) equilibrated with 20 mM HEPES (pH 7.6), 250 mM KCl, and 10% glycerol for further purification by size exclusion chromatography. The purity of the sample was analyzed on a 12% SDS-PAGE gel, and the concentration was estimated using Bradford reagent (BioRad, Inc., Hercules, CA, USA) (Figure S1). 

### 2.7. In Vitro CoAlation of AgrA

A total of 20 μL of reduced AgrA (40 μM) was incubated with 5 μL of 600 μM coenzyme A (CoA, C3019- Sigma Aldrich) for 10 min to allow CoA binding. Subsequently, 5 μL of 1.8 mM H_2_O_2_ was added to the reaction mixture and incubated for half an hour at 25 °C. The reaction mixture was passed through a MicroBiospin^TM^ 6 column to remove excess CoA and H_2_O_2_ before SPR analysis or treated with 10 mM NEM for 5 min at RT before WB analysis with anti-CoA antibody. 

### 2.8. Surface Plasmon Resonance (SPR)

Interaction studies of AgrA and CoAlated AgrA with P2 and P3 promoter DNA were performed on a BIACORE 2000 instrument (Biacore, Uppsala, Sweden). Both P2 and P3 promoters (complementary sequences 5′ TAACAGTTAAGTATTTATTTCCTACAGTTAGGCA 3′ and 5′ TTCTTAACTAGTCGTTTTTTATTCTTAACTGTAA 3′, respectively) were biotinylated at the 5′ end (Sigma Aldrich, Co.) and annealed with the unlabeled complementary strand prior to immobilization on a Streptavidin (SA) sensor chip (GE Healthcare). For annealing, an equimolar concentration of labelled and unlabeled DNA strands was dissolved in 1X Saline Sodium Citrate (1X SSC) buffer and denatured at 100 °C for 10 min in a water bath and gradually annealed to room temperature. A total of 1 μM of the hybridized promoters were immobilized on the flow cells (P1 in channel 2, P2 in channel 3) using 10 mM sodium acetate (pH 4.0), and the chip was primed with 1X PBST (1X PBS with 0.05% Tween 20). Approximately 500 RUs (response difference units) of each promoter were immobilized. The first flow cell (channel 1) in the sensor chip was used as the reference. The interaction experiments were performed in a flow buffer containing 20 mM HEPES (pH 7.6), 250 mM KCl, and 10% glycerol. A total of 50 µL of the substrate (AgrA or CoAlated AgrA) at various concentrations were passed over the flow cells (flow rate: 30 µL/min) and allowed to dissociate for 200 s. The sensor surface was regenerated with multiple injections of 0.05–0.1% SDS whenever required. The normalized response curves (reference subtracted) obtained were evaluated using BIA evaluation software. The data obtained were fit to a Langmuir 1:1 interaction model to obtain the rates of association (K_a_); dissociation (K_d_); and the equilibrium dissociation constant, K_D_ (K_d_/K_a_) (Table 1). The consistency between multiple datasets (performed with different protein preparations) was evaluated by comparing the values of theoretically fitted K_d_ and calculated K_d_ using BIA evaluation software.

## 3. Results

### 3.1. Identification of Cysteine Residues Involved in the CoAlation of AgrA

We have previously described protein CoAlation as a widespread and reversible PTM in bacteria and have identified numerous CoAlated proteins in response to oxidative and metabolic stress in *E. coli*, *B. megaterium*, and *S. aureus* [17]. Diamide induces oxidative disulfide exchange in cells and has been commonly used for studying oxidative stress responses in prokaryotic and eukaryotic cells. In diamide-treated *S. aureus*, 365 proteins were found to be CoAlated, which corresponded to ≈12% of the predicted proteome. Notably, 7% of CoAlated proteins were transcriptional regulators and some of them are involved in redox-sensing and the antioxidant response. These include the well-studied SarR, CtsR, PerR, SarS, and AgrA [17]. The regulation of AgrA by CoA was of particular interest to us because AgrA is uniquely positioned to sense and transduce signals from the environment, including nutrient availability and oxidative stress. CoA is a central metabolite for both anabolic and catabolic processes (such as amino acid metabolism, as well as fatty acid synthesis and oxidation), and its levels are controlled by nutrients, hormones, metabolites, and cellular stresses [22,23,24,25]. Moreover, the CoA thioester, acetyl-CoA, is required for the regulation of gene expression via protein acetylation. In addition, it was previously shown that CoAlation can modulate the function of key metabolic and antioxidant enzymes [17,18]. We were therefore interested to further investigate the modulation of the transcription factor AgrA by CoA.

**Figure 1:**
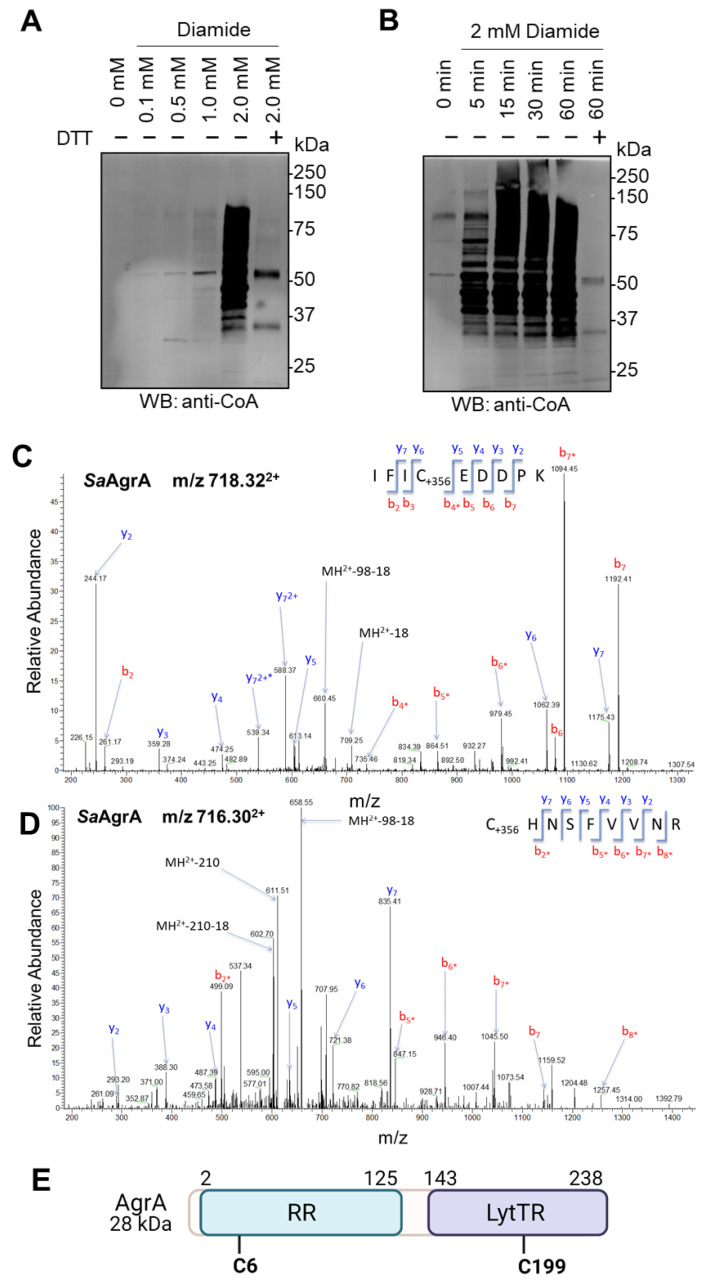
AgrA is CoAlated at Cys6 and Cys199 in diamide-treated *S. aureus*. (**A**) Anti-CoA Western blot analysis of protein CoAlation in *S. aureus* in response to a dose-course treatment with diamide. (**B**) Anti-CoA Western blot analysis of protein CoAlation in *S. aureus* in response to a time-course treatment with diamide (2 mM). To demonstrate that the protein-CoA binding involves a reversible disulfide bond formation, DTT (200 mM final) was added to protein extracts before SDS-PAGE analysis. Figures shown are representative of at least 3 independent repeats. (**C**,**D**) Mass spectra of CoAlated peptides corresponding to AgrA from the LC–MS/MS analysis of diamide-treated *S. aureus* cells. Sites of CoAlation were identified as Cys6—ILIC^6^EDDPK (**C**) and Cys199—C^199^HNSFVVNR (**D**) of AgrA. The asterisks (*) denote the loss of phosphoric acid (−98 Da) from the precursor and/or product ions that contained the CoA-modified cysteine residue. (**E**) Schematic diagram of AgrA domain organization with the location of CoA-modified cysteines indicated. RR—response regulatory domain; LytTR—DNA-binding domain. (Created with BioRender.com (accessed on 21 May 2021)).

Initially, we analyzed the pattern of protein CoAlation in *S. aureus* treated with diamide under various experimental conditions. Cells were grown to mid-log phase (OD_600_ = 0.7) in rich LB medium at 37 °C and treated with a dose- (Figure 1A) or time-course (Figure 1B) of diamide. Protein extracts were prepared as described in the Materials and Methods section, separated under non-reducing conditions, and analyzed by WB with anti-CoA monoclonal antibody. The results shown in Figure 1 revealed a weak immunoreactive signal in control (non-treated) samples, while diamide induced a significant increase in the number of immunoreactive bands in both a concentration- and time-dependent manner. Addition of DTT in the loading buffer resulted in the disappearance of most immunoreactive bands on the anti-CoA WB, confirming that diamide-induced CoA binding to proteins results from reversible disulfide bond formation.

To confirm the modification of AgrA by CoA and to map the site(s) of CoAlation, protein extracts from diamide-treated *S. aureus* (2 mM, 30 min) were digested with trypsin/LysC and analyzed by liquid chromatography tandem mass spectrometry (LC–MS/MS) as previously described [16]. Figure 1C,D shows the LC–MS/MS spectra of cysteine-containing CoAlated peptides derived from AgrA. Notably, Cys6 and Cys199 were found with an additional 356 Da, corresponding to covalently attached 4-phosphopantetheine. The position of CoA-modified cysteines relative to the overall structure of AgrA is schematically presented in Figure 1E, noting the position of oxidation-sensing Cys199 in the DNA-binding domain of AgrA.

### 3.2. Oxidizing Agents Induce AgrA CoAlation

The identification of CoA-modified AgrA in diamide-treated *S. aureus* by LC–MS/MS prompted us to investigate whether other types of oxidizing conditions might also induce AgrA CoAlation. 

**Figure 2:**
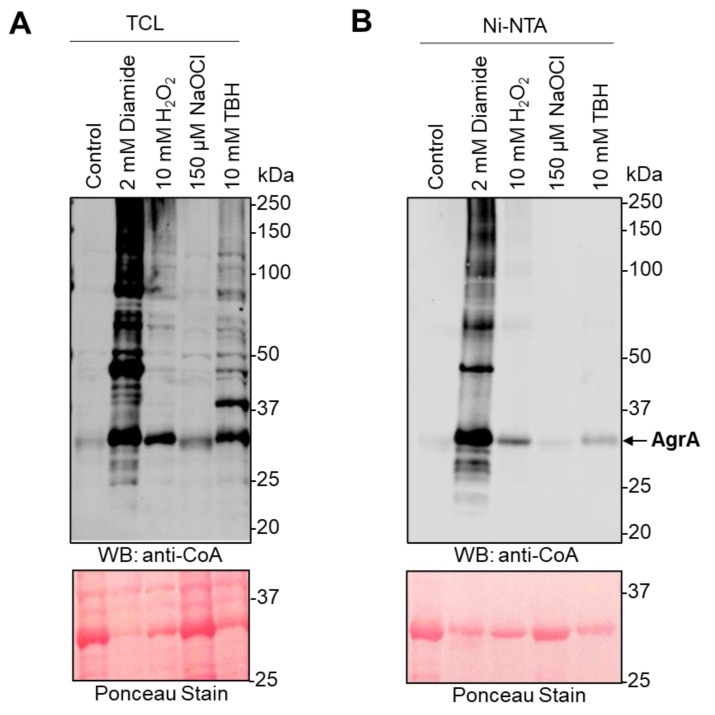
*Sa*AgrA CoAlation is induced by treatment with oxidizing agents. The expression of *Sa*AgrA in *E. coli* transformed with the pET28/His-*Sa*AgrA plasmid was induced with 0.1 mM IPTG for 20 min at 37 °C. Bacterial cultures were then treated with 2 mM diamide, 10 mM H_2_O_2_, 100 µM NaOCl, or10 mM TBH for 30 min. Harvested cell lysates were incubated with Ni-NTA Sepharose beads and *Sa*AgrA was pulled down. The total cell lysates (TCL) (**A**) and pulled down *Sa*AgrA (**B**) were analyzed by anti-CoA Western blots. The Ponceau stains served as loading control. The figures are representative of at least three independent repeats.

In this study, *E. coli* cells were transformed with the pET28/*Sa*AgrA plasmid, which drives the expression of N-terminally 6xHis-tagged *S. aureus* AgrA (*Sa*AgrA). Expression was induced with IPTG (20 min, 37 °C) as described in the Materials and Methods, and the culture was treated with 2 mM diamide, 10 mM H_2_O_2_, 100 µM NaOCl, or 10 mM *tert*-butyl hydroperoxide (TBH) for 30 min to induce oxidative stress. Overexpressed *Sa*AgrA was pulled-down from cell lysates using Ni^2+^-NTA Sepharose beads, and both total cell lysates (TCL) and the pulled-down proteins were analyzed by non-reducing SDS-PAGE followed by an anti-CoA WB. Figure 2A shows a significant induction of protein CoAlation in response to diamide treatment compared to control, confirming our previous results (Figure 1A,B). Strong immunoreactive bands are also visible in TBH- and H_2_O_2_-treated *E. coli*, whereas the weakest signal appears in response to NaOCl treatment. Immunoblotting of pulled-down *Sa*AgrA reveals a very faint immunoreactive signal in control cells, indicating that *Sa*AgrA exists predominantly in the non-CoAlated form in control conditions. Diamide treatment induces a significant increase (≈10-fold, Figure S2A) in the signal intensity of pulled-down *Sa*AgrA. Notably, the immunoreactivity appears across the full lane, possibly reflecting a high level of CoAlated protein monomers, dimers, and potentially oligomers. The high immunoreactivity might also represent the formation of covalent complexes between CoAlated AgrA and other proteins (some of which are CoA-modified). Overexpressed *Sa*AgrA is also strongly CoAlated after H_2_O_2_ and TBH treatments (four- and three-fold increase in signal intensity, respectively; Figure S2A), while only a weak signal is observed in the protein purified from NaOCl-treated cells (0.3-fold increase in signal intensity; Figure S2A).

### 3.3. AgrA CoAlation Is Induced by Glucose Deprivation 

Although bacteria can use a variety of sugars as a carbon source for energy production, glucose is the preferred choice. We have previously uncovered that glucose deprivation induces specific S-thiolation of redox-sensitive cysteine residues by CoA in bacteria, resulting in the formation of protein-CoA mixed disulfides (CoAlation) [17]. Furthermore, it was shown that protein CoAlation is a reversible modification that disappears in a time-dependent manner after the recovery of cells from glucose deprivation-induced stress. We were therefore interested to investigate whether AgrA was subject to the same modification by CoA during nutrient starvation. 

**Figure 3:**
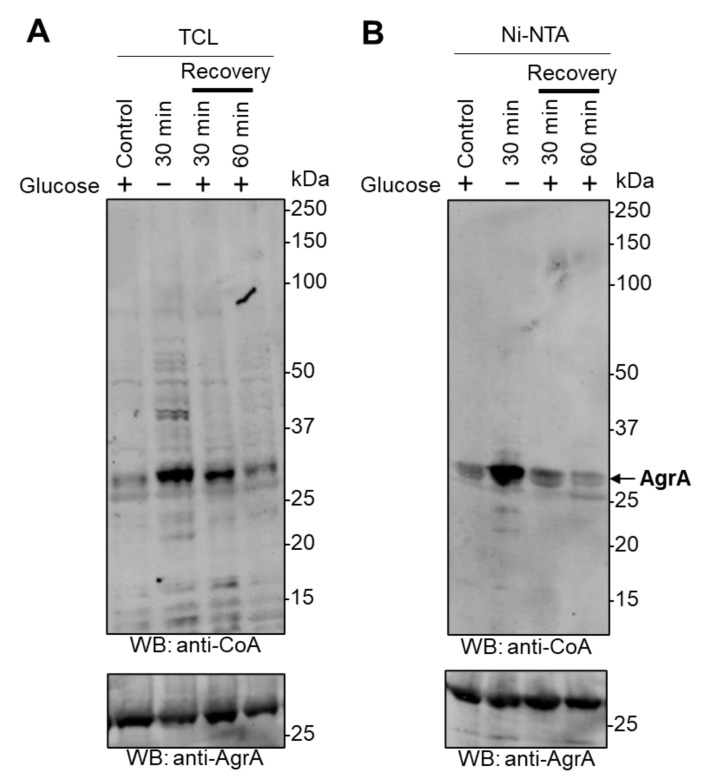
Glucose deprivation induces CoAlation of *Sa*AgrA in *E. coli*. The expression of *Sa*AgrA was induced with 0.1 mM IPTG for 20 min at 37 °C. Cells were then transferred and cultured in medium lacking glucose or any other source of carbohydrates for 30 min. The cultures of glucose-starved bacteria were then supplemented with 20 mM glucose and incubated at 37 °C for the indicated times to allow recovery. (**A**) Protein CoAlation in total protein extracts was examined by anti-CoA Western blot. (**B**) Overexpressed *Sa*AgrA was purified using Ni-NTA Sepharose and analyzed by Western blotting with anti-CoA antibody. The respective amounts of *Sa*AgrA are shown by Western blot with anti-AgrA antibodies. The figures are representative of at least three independent repeats.

In this study, recombinant 6xHis-tagged *Sa*AgrA was overexpressed in *E. coli* cells. Expression was induced with 0.1 mM IPTG and the culture grown in nutrient-rich LB medium for 20 min at 37 °C. Glucose deprivation stress was induced by transferring the culture to a M9 minimal medium lacking glucose or any other carbohydrate source for 30 min at 37 °C. To investigate whether CoAlation is reversible, we re-introduced glucose-starved *E. coli* overexpressing *Sa*AgrA to M9 minimal medium containing 20 mM glucose, and cells were grown for a further 30 min or 60 min at 37 °C. Cells were then lysed, and total protein extracts and affinity-purified *Sa*AgrA were analyzed by immunoblotting with anti-CoA and anti-AgrA antibodies. As shown in Figure 3A, there is a significant increase in the number and immunoreactivity of CoAlated proteins after glucose starvation, indicating that glucose deprivation induces the modification of proteins by CoA, which is consistent with our previously published findings [17]. Notably, the strongest signal intensity is seen at around 30 kDa, which corresponds to the molecular weight of overexpressed *Sa*AgrA. The same trend was observed in the analysis of total protein extracts on Figure 2A and was likely due to the fact that *Sa*AgrA levels are significantly higher than those of other proteins. Supplementing the glucose-deprived bacterial culture medium with glucose results in a time-dependent deCoAlation of cellular proteins, and the anti-CoA immunoreactive signal was similar to that seen in control cells after 60 min of recovery (Figure 3A). The anti-AgrA WB on Figure 3B (bottom panel) shows similar levels of pulled-down *Sa*AgrA in all examined samples. Glucose starvation induced a 2.9-fold increase in the anti-CoA signal intensity of overexpressed *Sa*AgrA when compared to control (Figure 3B and Figure S2B). Notably, reintroduction of glucose to the culture medium of starved cells leads to a gradual decrease in the CoAlation signal intensity, reaching similar levels as seen in control cells after 60 min of recovery (Figure 3B and Figure S2B).

### 3.4. Nitrogen Deprivation Induces AgrA CoAlation

Aside from carbon deprivation, nitrogen deprivation is another widely used method for inducing stress responses in bacteria. We were interested in examining whether nitrogen deprivation could lead to the modification of cellular proteins by CoA in a reversible manner, as well as in studying the mechanism of *Sa*AgrA CoAlation in response to nitrogen deprivation-induced stress.

**Figure 4:**
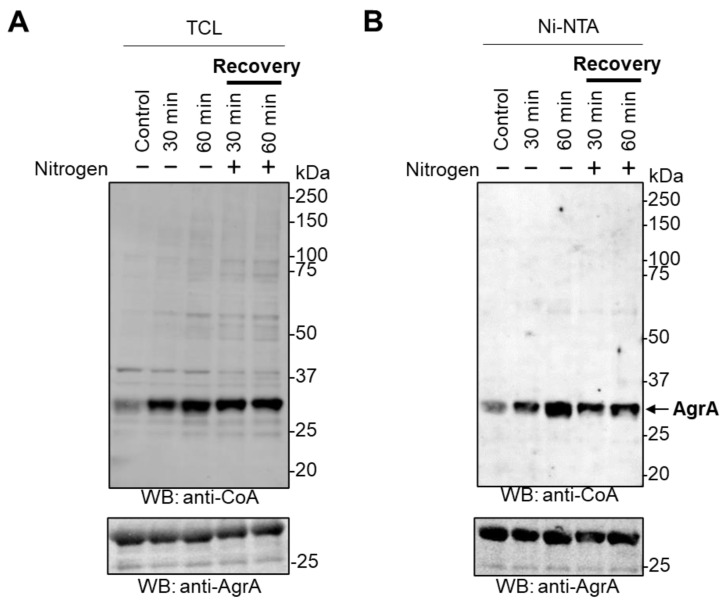
Nitrogen deprivation induces CoAlation of *Sa*AgrA in *E. coli*. Nitrogen-deprived cultures of *E.* *coli* cells transformed with the pET28/His-*Sa*AgrA were cultured in Gutnick minimal medium lacking a source of nitrogen for 30 or 60 min. They were then re-supplemented with 10 mM NH_4_Cl as the sole nitrogen source and incubated at 37 °C for the indicated times. CoAlation in total protein extracts (**A**) or of Ni-NTA pulled-down *Sa*AgrA (**B**) was examined by anti-CoA Western blot. The respective amounts of *Sa*AgrA are shown by Western blot with anti-AgrA antibodies. The figures shown are representative of at least three independent repeats.

*E. coli* cells transformed with the pET28/*Sa*AgrA plasmid were exposed to nitrogen starvation as described in the Materials and Methods. The nitrogen-deprived cultures were then re-supplemented with 10 mM NH_4_Cl and grown for an additional 30 min or 60 min at 37 °C to recover from stress. Total protein lysates and overexpressed *Sa*AgrA were extracted from bacteria as described in the Materials and Methods, and analyzed by anti-CoA and anti-AgrA WB. There is little or no immunoreactive signal in control *E. coli* cells overexpressing *Sa*AgrA (Figure 4A), which is in line with our previous observations (Figure 2A and Figure 3A). 

Culturing cells in nitrogen-deprived medium resulted in an upregulation of protein CoAlation, as numerous bands became apparent on the anti-CoA WB, most notably at ≈30 kDa, corresponding to the MW of overexpressed *Sa*AgrA (Figure 4A). Notably, the re-supplementation of nitrogen to starved cells did not result in deCoAlation of cellular proteins, as the same immunoreactive signal intensity is maintained even after 60 min of recovery in full media. Overexpressed *Sa*AgrA was pulled down from lysed cells and analyzed by immunoblotting with anti-CoA and anti-AgrA antibodies (Figure 4B). A relatively weak signal appears on the anti-CoA WB of *Sa*AgrA purified from control cells, as per our previous remarks (Figure 2B and Figure 3B). Notably, CoAlation of overexpressed *Sa*AgrA is strongly increased upon nitrogen starvation in a time-dependent manner, as the signal intensity increased by 2.1-fold after 30 min of starvation compared to control, and further to 3.2-fold after 60 min of starvation (Figure 4B and Figure S2C). The same trend was observed with pulled-down *Sa*AgrA as with total protein extracts during the recovery of cells from nitrogen starvation, revealing no significant reduction of signal intensity as *Sa*AgrA remained strongly CoAlated even after 60 min of recovery (2.7-fold increase compared to control). The efficient deCoAlation of cellular proteins and overexpressed *Sa*AgrA after the recovery of cells from glucose deprivation, as well as a lack of deCoAlation after nitrogen deprivation, may point to different regulatory mechanisms operating in response to carbon compared to nitrogen starvation.

### 3.5. In Vitro AgrA CoAlation Inhibits its DNA-binding Activity

Previous findings showed that CoAlation affects the activity of metabolic and signaling enzymes in a DTT-dependent manner, including creatine kinase, peroxiredoxin 5, glyceraldehyde 3-phosphate dehydrogenase (GAPDH), and Aurora A kinase, among others [16,17,18,19]. Furthermore, CoAlation of *S. aureus* GAPDH was shown to protect the catalytic cysteine 151 from overoxidation and irreversible inactivation [17,18]. The LC–MS/MS analysis revealed that *Sa*AgrA is CoAlated on Cys6 and Cys199 in *S. aureus* under oxidative stress (Figure 1B,C), and previous studies have suggested that Cys199 is crucial for the redox-sensing mechanism of AgrA in *S. aureus* [5]. In an effort to understand whether CoAlated Cys199 in the DNA-binding domain of AgrA alters its interaction with DNA, we evaluated the binding of native and CoAlated AgrA to the P2 and P3 promoters by surface plasmon resonance (SPR).

**Figure 5:**
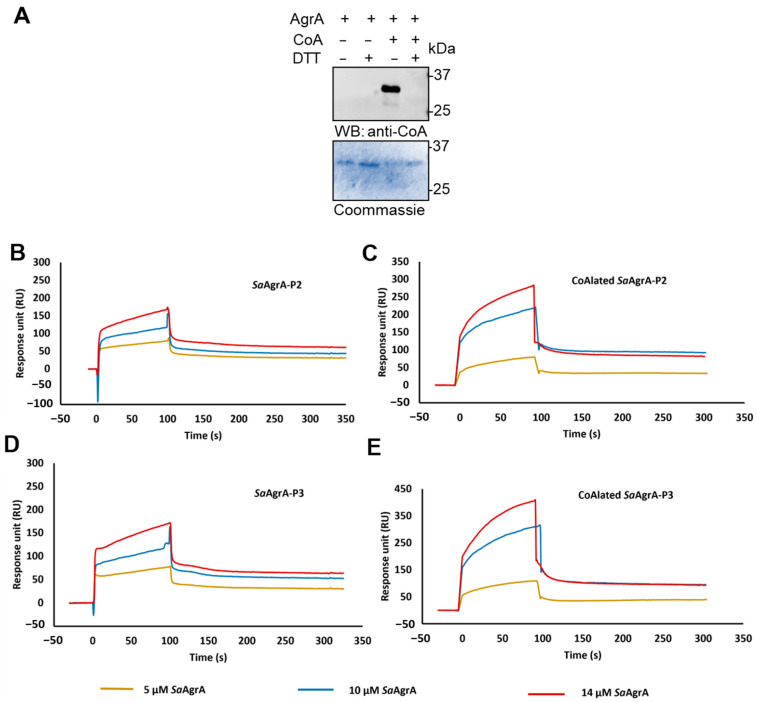
CoAlation of recombinant *Sa*AgrA differentially affects its DNA-binding activity to P2 and P3 promoters. (**A**) In vitro CoAlation of recombinant *Sa*AgrA was analyzed by anti-CoA Western blot. Immediately after in vitro CoAlation, interaction of *Sa*AgrA and CoAlated *Sa*AgrA to P2 and P3 promoters was analyzed by surface plasmon resonance (SPR). (**B**,**C**) Binding profiles of *Sa*AgrA and CoAlated SaAgrA to the P2 promoter are shown. (**D**,**E**) Binding profiles of *Sa*AgrA and CoAlated *Sa*AgrA to the P3 promoter are shown. Interaction parameters derived from the sensorgrams are compiled in Table 1. The flow and sample buffer maintained was 20 mM HEPES (pH 7.6), 250 mM KCl, and 10% glycerol.

To produce CoAlated recombinant AgrA for use in these studies, 6xHis-tagged *Sa*AgrA was expressed and purified from *E. coli* using Ni-NTA sepharose, as described in the Materials and Methods section (Figure S1). CoAlated *Sa*AgrA was prepared using a previously established in vitro CoAlation assay by incubating recombinant *Sa*AgrA in the presence of CoA for 30 min at RT. Immunoblotting the reaction mixture with anti-CoA antibody revealed a strong immunoreactive signal at ≈30 kDa, corresponding to CoAlated recombinant *Sa*AgrA (Figure 5A). Reducing conditions (100 mM DTT) completely abolished the signal, confirming that the modification of recombinant *Sa*AgrA by CoA occurrs via a disulfide bond. The kinetics of *Sa*AgrA and CoAlated *Sa*AgrA binding with the promoters P2 and P3 are compiled in Table 1. As is evident from the sensorgrams (Figure 5B−E), CoAlation substantially influences the DNA-binding ability of *Sa*AgrA. We noted an approximately 10-fold reduction in the binding affinity of CoAlated *Sa*AgrA with the P2 promoter (Table 1). In the case of the P3 promoter, the DNA binding was altered even more significantly (Table 1, Figure 5D,E).

The kinetic parameters (Table 1) revealed that CoAlation substantially influences the DNA-binding ability of *Sa*AgrA towards both P2 and P3 promoters. While an approximate 10-fold reduction in the binding affinity of CoAlated *Sa*AgrA to the P2 promoter was noted, CoAlation influenced *Sa*AgrA interactions with the P3 promoter even more significantly. It is worth noting in this context that the P3 promoter was sub-optimal (20 bp spacing between the –35 and −10 promoter elements) when compared to the P2 promoter. Indeed, the transcription competent open promoter complex (RPo) occurrs more readily at P2 than at P3 [21]. The finding that CoAlation abrogates *Sa*AgrA-P3 interactions is thus consistent with previous observations that expression of RNAIII, a pleiotropic effector involved in the upregulation of exotoxins such as alpha-haemolysin, is tightly regulated.

## 4. Discussion

Bacterial cells use various strategies to maintain redox homeostasis, including transcriptional regulation, which allows for the expression of genes involved in antioxidant defense [26]. Redox-sensing regulators in bacteria respond to diverse environmental cues such as the availability of nutrients and oxygen, as well as exposure to reactive oxygen and nitrogen species, among others. These redox signals are transduced by transcriptional regulators through specific mechanisms, involving upregulated expression of low molecular weight thiols, antioxidant enzymes, and detoxifying proteins [27,28,29]. *S. aureus* is an aggressive opportunistic pathogen due to its prominent virulence and antibiotic resistance, which is achieved through adaptive and timely coordination of gene expression for virulence, growth, and survival [2]. These include two-component regulatory systems, transcription factors, and regulatory RNAs. *S. aureus* expresses over 250 sRNA genes, many of which are responsible for the adaptation to environmental changes including oxidative and metabolic stress conditions. The *agr* two-component system is perhaps the most-studied and has well elucidated roles in quorum-sensing; however, much less is known about its role in oxidation sensing [1]. 

The recent development of an LC–MS/MS methodology and a specific anti-CoA antibody allowed for a proteome-wide CoAlome analysis in mammalian and bacterial cells, which identified over 2100 CoAlated proteins in response to oxidative or metabolic stress. Functional classification of CoAlated proteins revealed that in contrast to mammalian cells, numerous transcription factors and regulators are found to be CoA-modified in bacteria [16,17]. The susceptibility of bacterial transcription factors to oxPTMs suggests their importance in the antioxidant response. The most studied oxPTM is glutathionylation, and it was previously shown to be involved in the modulation of bacterial virulence and the activity of transcriptional regulators [9,10]. However, GSH is not available as an antioxidant in *S. aureus*, which only produces two low molecular weight thiols, BSH and CoA. BSH is considered a key protective thiol in *S. aureus* antioxidant defense by forming protein–BSH mixed disulfides through bacillithiolation [30,31]. While there are no reports to our knowledge describing bacillithiolation of AgrA during oxidative stress, it has been found that AgrA possesses a redox-sensitive cysteine residue that is a target of oxidative stress response, and therefore susceptible to oxPTMs. Cys199, located in the DNA-binding domain of AgrA, was identified as the oxidation-sensing residue, and its oxidation was shown to inhibit the DNA-binding activity of AgrA [5]. Furthermore, another study revealed that Cys199 of *S. aureus* AgrA (among other cysteine residues) is a substrate for S-nitrosylation, and this modification was shown to inhibit *agr* transcription [32]. 

**Figure 6:**
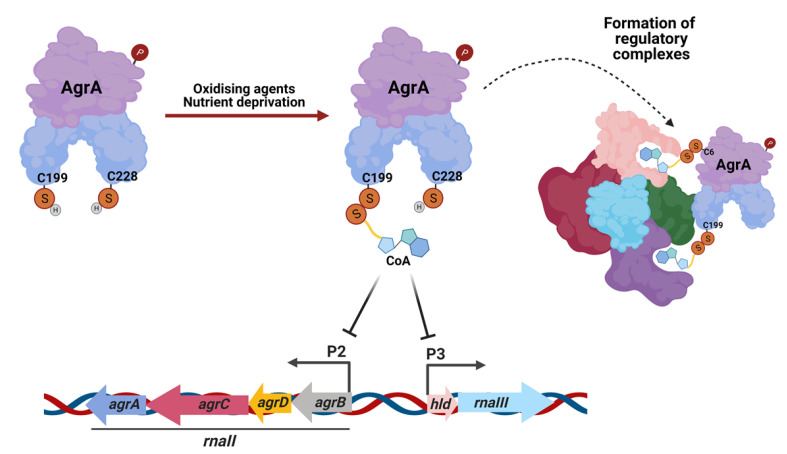
Schematic representation of the effect of AgrA CoAlation on DNA binding at P2 and P3 promoters. Upon oxidative stress or nutrient deprivation, AgrA is CoAlated at Cys199, which inhibits its DNA-binding to P2 and P3 promoters. CoAlation of AgrA may promote the formation of regulatory complexes through the recognition of the ADP moiety of CoA by proteins containing the Rossmann binding fold, which might be involved in the activation of antioxidant response elements. (Created with BioRender.com (accessed on 21 May 2021)).

In the present study, we reveal the induction of AgrA CoAlation in response to a panel of oxidizing agents (diamide, H_2_O_2_, and TBH) and metabolic stress induced by glucose or nitrogen deprivation. The sites of CoAlation were mapped to Cys199 and Cys6 using LC–MS/MS. Since Cys199 is the redox-sensitive residue located in the DNA-binding region, we hypothesized that CoAlation could interfere with DNA binding. Indeed, SPR analysis showed that CoA-modified AgrA had significantly lower affinity towards the P2 and P3 promoters than non-CoAlated AgrA. Active AgrA triggers transcription from its own operon (agrBDCA, i.e., the P2 promoter), as well as the divergently transcribed regulatory RNAIII (i.e., the P3 promoter). Dissociation of CoAlated AgrA from its promoters would therefore result in decreased transcription of AgrB, AgrD, AgrC, and AgrA, as well as the downregulation of RNAIII expression (Figure 6). 

It was previously shown in a microarray study that active AgrA downregulates transcription of the *S. aureus* GSH peroxidase gene encoding for *BsaA*, a key enzyme in bacterial resistance to oxidative stress [33]. Another study confirmed that AgrA represses *BsaA* expression, suggesting that it occurs via a direct DNA-binding mechanism [5]. Interestingly, oxidative stress was shown to relieve the AgrA-mediated downregulation of *BsaA* expression. Similarly, oxidative- or metabolic stress-induced CoAlation of AgrA might result in de-repression of *BsaA* transcription and allow for efficient bacterial antioxidant defense. Dissociation of CoAlated AgrA from the P2 promoter might constitute an adaptive response in bacteria to initiate antioxidant defense, revealing the protective role of CoAlation in *S. aureus*.

Protein CoAlation was described as a reversible PTM in bacteria and mammalian cells where numerous proteins were shown to be efficiently deCoAlated after the removal of oxidizing agents or metabolic stress [16,17]. Data presented in this study reveal that AgrA CoAlation is reversible after the recovery of cells from glucose starvation-induced metabolic stress, which was also the case for numerous proteins in the total cell lysates (Figure 3). This is consistent with previous findings which showed that protein CoAlation in bacteria (*E. coli*, *B. megaterium*, and *S. aureus*) exposed to oxidative or metabolic stress is efficiently reversed after the recovery of cells in oxidant-free media. Previous studies described the effects of CoAlation on metabolic enzymes in bacteria and mammalian cells. Numerous enzymes including creatine kinase, peroxiredoxin 5, GAPDH, and Aurora A kinase were found to be CoAlated in response to oxidative or metabolic stress, and CoAlation was shown to affect their activity and protect them from irreversible overoxidation [19,34,35]. It was also proposed that CoAlation might serve as a scaffold for the formation of regulatory interactions and complexes. Since CoA is a relatively large molecule comprising of a pantetheine tail and an ADP moiety, protein CoAlation might form novel binding sites, specifically for proteins containing the Rossmann fold that could recognize the ADP moiety of CoA. In the case of AgrA, covalent modification by CoA under oxidative or metabolic stress may allow for the formation of regulatory interactions implicated in the transduction of redox signaling and antioxidant gene expression (Figure 6).

## 5. Conclusions

Altogether, this study provides insight into a novel mode of AgrA regulation by a key metabolic regulator, CoA. Generated results strengthen the previously established link between the quorum-sensing and oxidation-sensing role of the *agr* system via the oxidation-sensitive Cys199 on AgrA. The inhibitory effect of AgrA CoAlation on the activity of a quorum sensing transcription factor is of particular interest. It is well known that both nutrient deprivation and oxidative stress lead to the reduction of metabolically active CoA thioesters and the increase in the reduced form of CoA. The increase in the CoA: CoA thioesters ratio is an essential prerequisite for the involvement of CoA in the antioxidant defense via protein CoAlation. Future studies may explore the potential role of CoAlation in regulation of other transcription factors implicated in redox regulation in prokaryotic and eukaryotic cells. The emerging function of protein CoAlation in redox regulation will be the focus of future studies in pathologies associated with oxidative stress, including cancer, neurodegeneration, and ischemia/reperfusion injury.

## Figures and Tables

**Table 1:** Interaction parameters of CoAlated AgrA to P2 and P3 promoters.

Promoter	Analyte	k_a_ (1/Ms)	k_d_ × 10^−2^ (1/s)	K_D_ (μM)
P2	AgrA	5.75 × 10^3^	1.01	1.75 ± 0.58
	CoAlated AgrA	9.54 × 10^2^	1.34	14.1 ± 7.52
P3	AgrA	6.44 × 10^4^	2.12	0.33 ± 0.03
	CoAlated AgrA	1.97 × 10^−2^	7.37	(3.74 ± 1.19) × 10^6^

## Data Availability

Not applicable.

## Supplementary Materials

The following are available online at https://www.mdpi.com/article/10.3390/antiox10060841/s1, Figure S1: Purification of recombinant 6XHis-SaAgrA. Figure S2: Quantitation of the WB signal intensity in treated and untreated samples of CoAlated SaAgrA.
