# Supplementary material for: Redox Regulation of the Quorum-sensing Transcription Factor AgrA by Coenzyme A
Source: Antioxidants (Basel). 2021 May 25;10(6):841. doi: 10.3390/antiox10060841 (PMC8228455; doi:10.3390/antiox10060841)
Supplement: Supplementary file 1 [file antioxidants-10-00841-s001.zip › antioxidants-1217684-supplementary.pdf]

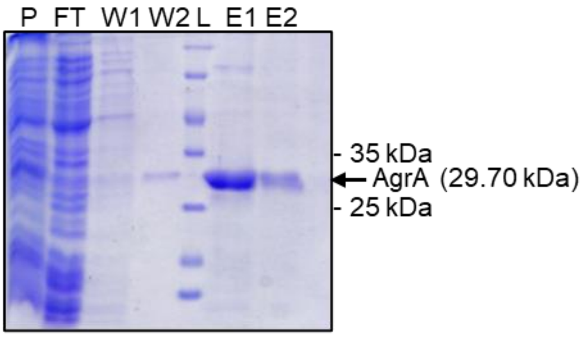

Supplementary Figure 1. Purification of recombinant 6XHis-*Sa*AgrA. P- Pellet, FT- Flow Through, W – Wash, Elutions- E1 (100 mM imidazole) and E2 (250 mM imidazole), L- Protein Ladder.

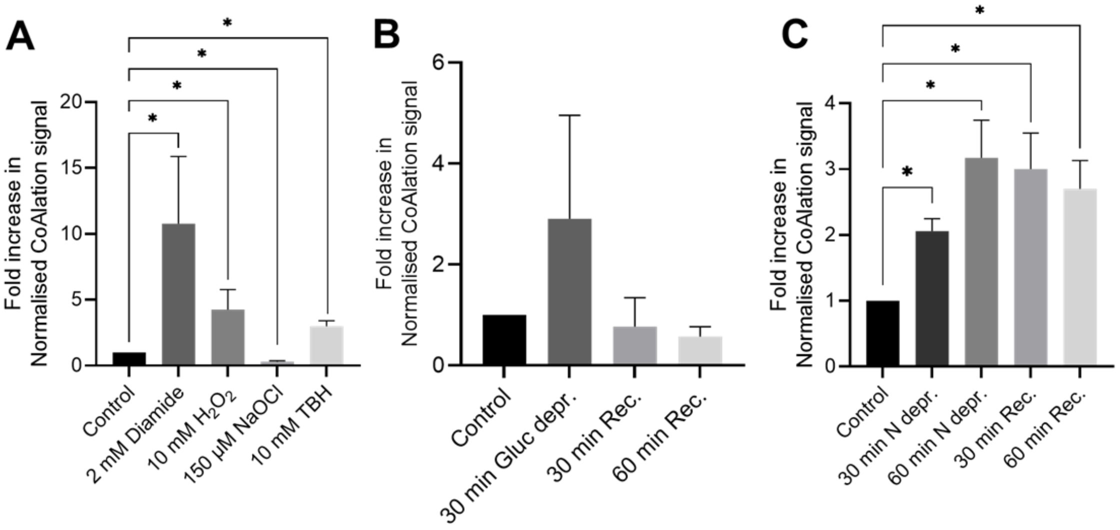

Supplementary Figure 2. Quantitation of the WB signal intensity in treated and untreated samples of CoAlated *Sa*AgrA. Band intensities for each sample on the anti-CoA WB were normalized with pulled down SaAgrA intensity on the anti-AgrA WB. **(A)** Plotted bar graph of mean fold-increase in *Sa*AgrA CoAlation from bacterial cells treated with 2 mM diamide, 10 mM H_2O_2, 150 µM NaOCl and 10 mM TBH. Quantitative data is representative of Figure 2B. Diamide = 10.7; H_2O_2 = 4.3; NaOCl = 0.31; TBH = 3.0. **(B)** Plotted bar graph of mean fold-increase in *Sa*AgrA CoAlation from bacterial cells subjected to 30 min glucose deprivation (gluc depr.) or resupplemented with glucose (rec.) for 30 and 60 min. Quantitative data is representative of Figure 3B. Gluc depr. = 2.9; 30 min Rec. = 0.77; 60 min Rec.= 0.57. **(C)** Plotted bar graph of mean fold-increase in *Sa*AgrA CoAlation from bacterial cells subjected to 30 min and 60 min of nitrogen deprivation (N depr.) or resupplemented with nitrogen (Rec.) for 30 and 60 min. Quantitative data is representative of Figure 4B. 30 min N depr. = 2.05; 60 min N depr.. = 3.17; 30 min Rec.= 3.0; 60 min Rec.= 2.7. Data represent mean ± SEM from n = 2 or 3 experiments. p values were calculated using ratio paired t-tests of each treatment condition compared to control (* $p < 0.05$).
